# Simplified rapid low-dose buprenorphine induction method for individuals using fentanyl: a case series

**DOI:** 10.1186/s13722-025-00620-8

**Published:** 2025-11-06

**Authors:** Ryan Alexander, Noah Woford

**Affiliations:** 1 McNabb Center, 5310 Ball Camp Pike, Knoxville, TN 37921 USA; 2 University of Tennessee Graduate School of Medicine, 1924 Alcoa Hwy., Knoxville, TN 37920 USA; 3 Lincoln Memorial University – DeBusk College of Osteopathic Medicine, 6965 Cumberland Gap Pkwy, Harrogate, TN 37752 UK

**Keywords:** Opioid use disorder, Medication for opioid use disorder, Buprenorphine, Detox, Low dose induction, Precipitated withdrawal

## Abstract

**Objectives:**

Among individuals with opioid use disorder using fentanyl, standard initial doses (2-4 mg) of buprenorphine may precipitate withdrawal, often preventing successful induction. Rapid low-dose induction is an emerging approach designed to mitigate this risk. This study describes a simplified rapid low-dose buprenorphine induction protocol in facilitating treatment initiation among patients presenting to an outpatient clinic.

**Methods:**

This case series includes chart review data from nine patients with opioid use disorder treated at an outpatient substance use clinic who were initiated on buprenorphine-naloxone maintenance therapy. All had recent fentanyl use confirmed by UDS. Patients were instructed to follow a simplified induction protocol consisting of 1 mg of buprenorphine-naloxone, via 1/8th of an 8 − 2 mg sublingual film, administered at home hourly for 8 h, followed by maintenance dosing of 8 mg twice daily. Patients were advised to wait at least 24 h since last fentanyl use prior to initiating the induction protocol. Successful induction was defined as being on maintenance treatment at a follow-up appointment one week later.

**Results:**

Of the nine patients who began the rapid low-dose induction protocol, seven successfully transitioned to buprenorphine-naloxone maintenance by the 7-day follow-up. Two patients did not return for follow-up.

**Conclusion:**

In this case series, the simplified rapid low-dose buprenorphine induction protocol was well-tolerated and 77.8% of patients using fentanyl were able to successfully initiate buprenorphine-naloxone maintenance. Benefits of this protocol are use of a single, standard dose of buprenorphine-naloxone, rapid induction timeline over only 8 h, and simple patient instructions.

## Introduction

The third and now fourth waves of the ongoing opioid epidemic in the United States have been characterized by the increasing prevalence of fentanyl and stimulants respectively in the unregulated drug supply [1]. Compared to other opioids, fentanyl’s lipophilic nature results in prolonged elimination, heightening the risk of precipitated withdrawal for several days after cessation [2]. In the setting of a fentanyl-predominant unregulated opioid drug supply [3, 4], avoiding buprenorphine-precipitated withdrawal has emerged as a central challenge in the treatment of opioid use disorder (OUD).

Initial buprenorphine induction methods were developed when non-fentanyl opioids dominated the illicit drug supply. As a result, standard induction strategies are often ineffective or harmful due to the increased risk of precipitated withdrawal associated with fentanyl [5].

To mitigate this risk, various alternative induction methods have been explored, including microdose (low-dose) and macrodose (high-dose) approaches [6, 7]. Microdose inductions present practical challenges: (1) the need for uncommon buprenorphine formulations (i.e. transdermal patch), (2) use of multiple formulations (i.e. transdermal and sublingual), (3) complex dosing transitions, and (4) prolonged protocols [8]. Additionally, these challenges vary between inpatient and outpatient settings, complicating implementation across care environments [9, 10].

In this report, we describe a simplified, and rapid low-dose induction (RLDI) protocol distinct from other low-dose techniques (e.g. 4-day and 7-day protocols [8]). This method has multiple practical benefits over typical microdosing that potentially make it more effective for use in the outpatient setting. First, this RLDI protocol uses a standard buprenorphine formulation (8 − 2 mg buprenorphine-naloxone sublingual film), which makes prescribing the initial rapid induction and subsequent daily maintenance doses simple and streamlined without having to prescribe multiple different doses or formulations of buprenorphine products. Second, this protocol omits concurrent opioid agonist dosing, further simplifying opioid dosing during induction. Third, this induction method was completed by patients at home thereby improving convenience for patients. Finally, this protocol completes induction within 8 h, which is markedly faster than other reported protocols [6]. We described this approach through a retrospective chart review in an outpatient clinical setting.

### Methods

This case series evaluated patients who underwent a novel, simplified RLDI protocol: 1/8 of an 8 − 2 mg buprenorphine-naloxone (BUP-NLX) sublingual film (1 mg) administered hourly for 8 h. All patients were using fentanyl, confirmed by fentanyl positive drug screen (UDS). Patients were advised to wait at least 24 h from last use and to initiate the protocol unobserved at home only after experiencing at least one objective withdrawal symptom (diaphoresis, piloerection, yawning, etc.). Importantly, patients were educated that they did not need to wait to develop severe opioid withdrawal syndrome prior to initiating the RLDI but did need to wait until they experienced some objective withdrawal findings. If patients experienced worsening withdrawal symptoms after any 1 mg dose, they were instructed to wait an additional hour prior to continuing the next 1 mg dose.

Patients were prescribed 14 BUP-NLX 8 − 2 mg sublingual films (one week supply) with instruction on how to divide the first dose into 1/8ths. The prescription was filled at an on-site pharmacy affiliated with the outpatient clinic where patients were being treated. The 8 − 2 mg films were divided by the patient into eight equal doses by folding and tearing/cutting the film. Patients were instructed to fold and cut the film in half, then cut the halves in half (quarters), then cut the quarters in half. Each of the resulting 8 pieces are approximately equal to 1 mg units. Upon completing the eight 1 mg doses, patients were instructed to start standard maintenance dosing of one BUP-NLX 8 − 2 mg sublingual film twice daily starting 8–12 h after completing RLDI. The combination product was used unless there was an allergy.

Cases were identified through a retrospective chart review of all new patients evaluated at an outpatient ‘safety net’ substance use treatment clinic in East Tennessee between January 1, 2024, and March 4, 2025. A data abstraction tool was created to include variables of interest and was used during chart review. Inclusion criteria were: (1) recent fentanyl use indicated by an immunoassay UDS, (2) agreement to initiate BUP-NLX maintenance, and (3) goal of abstinence from ongoing non-prescribed opioid use. Exclusion criteria were: (1) UDS positive for buprenorphine (due to pre-treatment and not requiring a new induction), (2) documented recent abstinence due to incarceration or hospitalization within the prior 7 days (eliminating risk of precipitated withdrawal) and (3) recent benzodiazepine or alcohol use (Due to possible confounding due to masking withdrawal symptoms). Successful induction was defined as returning to the one-week follow-up appointment while on BUP-NLX maintenance (confirmed by patient report and positive buprenorphine UDS). The study was reviewed and exempted as secondary reaserch by the McNabb Center Institutional Review Board.

## Results

Of 319 new patient charts reviewed, 13 met inclusion criteria and were recommended to start the RLDI. Of these 13, 4 patients were excluded from this report: 1 patient’s UDS was negative for fentanyl, 1 patient did not provide a UDS, 1 patient had 5 days of abstinence due to hospitalization prior to presenting to clinic, 1 patient reported his last use was 7 days prior. Overall 9 patients were included in the final analysis (Table 1). The mean age was 33.6 years; 5 of 9 (55.6%) were Male, and 7 of 9 (77.8%) reported injection drug use. Most patients were housed, unemployed, and uninsured or underinsured. All tested positive for fentanyl; additional substances detected included opiates (6 of 9, 66.7%) and methamphetamine (4 of 9, 44.4%). All patients were negative for buprenorphine, benzodiazepines, and alcohol at presentation.

Of the 9 patients initiating the RLDI protocol, 7 (77.8%) successfully completed induction and returned for their one-week follow up on BUP-NLX maintenance (Table 2).

**Table 1:** Characteristics at evaluation of 9 patients with Fentanyl use disorder initiated on a rapid low-Dose induction protocol in Knoxville, TN, 2024

	Patient								
	1	2	3	4	5	6	7	8	9
Age	34	31	31	31	29	23	34	41	48
Sex	M	M	F	M	F	F	F	F	M
Housing	sober living	own home	own home	own home	friends	friends	friends	own home	unstable
IVDU^1^	yes	yes	no	yes	yes	no	yes	yes	yes
Insurance	uninsured	uninsured	under-insured	uninsured	under-insured	medicaid	uninsured	uninsured	uninsured
Employed	no	yes	yes	yes	yes	no	no	no	no
**UDS (Evaluation)**									
Fentanyl	+	+	+	+	+	+	+	+	+
Amphetamines	+	-	-	-	+	-	-	+	-
Methamphetamine	+	-	-	-	+	-	-	+	+
Benzodiazepine	-	-	-	-	-	-	-	-	-
Buprenorphine	-	-	-	-	-	-	-	-	-
Cocaine	-	-	-	+	-	-	+	-	-
MDMA^2^	-	-	-	-	-	-	-	-	-
Opiates*	-	+	-	-	+	+	+	+	+
Tramadol	-	-	-	-	-	-	-	-	-
Alcohol	-	-	-	-	-	-	-	-	-
Methadone	-	-	-	-	-	-	-	-	-
THC^3^	+	-	+	-	-	-	-	+	+
Oxycodone	-	-	-	-	-	-	-	-	-

^1^IVDU: Intravenous Drug Use, ^2^MDMA: 3,4-Methylenedioxymethamphetamine (ecstasy), ^3^THC: Tetrahydrocannabinol (cannabis), *Opiate screen identifies morphine, heroin, codeine

**Table 2:** Characteristics at Follow-Up of 9 patients with Fentanyl use disorder initiated on a rapid low-dose induction protocol in Knoxville, TN 2024

Patient	1	2	3	4	5	6	7	8	9
Showed up to Follow-up Appointment	yes	yes	yes	yes	yes	no	no	yes	yes
Days to follow-up	7	7	3	7	7	n/a	n/a	8	7
Time since last use at initial evaluation (hrs)	20	24	12	12	18	8	6	36–48	48
Induction Challenges	none	none	none	nausea + vomiting	none	unknown	unknown	none	none
Referral Source	ED	Self	ED	ED	ED	ED	Self	Self	Self
Dose of BUP-NLX at Follow-up	16 mg	16 mg	16 mg	16 mg	16 mg	n/a	n/a	16 mg	16 mg
**UDS (Follow-up)**									
Fentanyl	+	-	+	+	+			-	+
Amphetamines	-	-	-	-	-			+	-
Methamphetamine	-	-	-	-	+			+	+
Benzodiazepine	-	-	-	-	-			-	-
Buprenorphine	+	+	+	+	+			+	+
Cocaine	-	-	-	-	-			-	-
MDMA^1^	-	-	-	-	-			-	-
Opiates*	-	-	-	-	-			-	+
Tramadol	-	-	-	-	-			-	-
Alcohol	-	-	-	-	-			+	-
Methadone	-	-	-	-	-			-	-
THC^2^	-	-	-	-	-			-	+
Oxycodone	-	-	-	-	-			-	-

^1^MDMA: 3,4-Methylenedioxymethamphetamine (ecstasy), ^2^THC: Tetrahydrocannabinol (cannabis), *Opiate screen identifies morphine, heroin, codeine

## Discussion

Successful initiation of BUP-NLX treatment is essential in combating the opioid epidemic. Given the dominance of fentanyl in the unregulated opioid supply, effective induction strategies that prevent precipitated withdrawal are increasingly critical. Precipitated withdrawal can result in serious physical and psychological consequences, potentially deterring patients from future buprenorphine treatment [7, 11]. In addition, it is important to reach therapeutic BUP-NLX dosing as quickly as possible to prevent return to use. This case series highlights a practical RLDI protocol for initiating BUP-NLX in people using fentanyl that combines simplicity and rapidity while avoiding precipitated withdrawal.

Previous research has shown mixed outcomes with induction protocols, especially in people who use fentanyl [8, 12]. The RLDI protocol described here reaches maintenance dosing in just 8 h, potentially improving patient tolerance, adherence, and retention due to the expedited nature. Its simplicity and use of a single, standard buprenorphine formulation (8 mg sublingual film) make it feasible in many settings, including outpatient clinics and emergency departments. The single dose formulation ensures that prescribers can easily send a single prescription for the standard dose of BUP-NLX without requiring multiple different prescriptions of different doses or formulations thereby reducing a barrier to initiation of treatment.

By utilizing a single BUP-NLX dose and formulation and simply dividing the first dose into eighths, instructing patients on how to complete this RLDI is quick and easy for patients to comprehend and remember thereby maximizing its clinical utility. Furthermore, pharmacies are more likely to stock, and have supply of, standard 8 mg BUP-NLX films as opposed to other doses and formulations. Lastly, this method may avoid overlapping full-agonist dosing during induction as is done with other overlapping low-dose induction methods.

While findings are promising, this study has multiple limitations. The small sample size restricts generalizability and may overstate effects due to random variation. Patient self-administration introduces variability in adherence and accuracy. We did not administer doses to patients and only followed up after one week. It is possible that patients did not follow the recommended dosing. However, it is reassuring that most patients who were advised to follow the RLDI protocol returned at one week successfully on maintenance treatment. In addition, ongoing unregulated opioid use cannot be ruled out by UDS results, however, patients denied ongoing unregulated opioid use.

In addition, although this RLDI protocol seeks to prevent precipitated withdrawal while also rapidly inducing patients on to BUP-NLX treatment, it still requires a period of abstinence and the development of some withdrawal symptoms prior to initiating. Only mild withdrawal is necessary prior to initiating the RLDI protocol but can be difficult for some patients and may prevent success. Although additional adjunctive medication treatments were not provided to this cohort of patients, it may be beneficial to offer such medications for some patients and may improve successful induction beyond what is seen in this sample of patients. Of note, all patients were negative for benzodiazepines, which could have acted as a confounder by masking withdrawal symptoms. However, one patient tested positive for alcohol at follow up but did not say they drank due to withdrawal symptoms.

Future research should employ larger cohorts and more controlled dosing conditions with more frequent follow-up. Additionally, regional variations in fentanyl supply may influence applicability in other geographic areas with a different illicit drug supply. Adjunctive medications for withdrawal symptoms may be useful to add to this simplified RLDI protocol.

## Data Availability

The datasets used and/or analyzed during the current study are available from the corresponding author on reasonable request.
